# Characterization of the whole chloroplast genome *Caulerpa lentillifera* J. Agardh (Bryopsidales, Chlorophyta)

**DOI:** 10.1080/23802359.2018.1524274

**Published:** 2018-10-31

**Authors:** Dahai Gao, Chaohua Huang, Jianting Yao, Yuhang Li, Wei Tan, Zhongmin Sun

**Affiliations:** a Department of Marine Organism Taxonomy and Phylogeny, Institute of Oceanology, Chinese Academy of Sciences, Qingdao, China;; b Guangxi Zhuang Autonomous Region Institute of Product Quality Inspection, Nanning, China;; c Key Laboratory of Experimental Marine Biology, Institute of Oceanology, Chinese Academy of Sciences, Qingdao, China;; d Academy of Marine and Fishery of Hainan Province, Haikou, China

**Keywords:** *Caulerpa lentillifera*, chloroplast genome, phylogenetic analysis

## Abstract

The whole chloroplast genome (cp DNA) sequence of *Caulerpa lentillifera* J. Agardh has been characterized from Illumina pair-end sequencing. The circular cpDNA was 119,402 bp in length, containing 122 genes, which included 91 protein-coding genes, 28 tRNA genes, and 3 ribosomal RNA genes (four rRNA species). The overall AT content of *C. lentillifera* cpDNA is 67.4%. The 48 genes phylogenetic analysis suggested that *C. lentillifera* formed a monophyletic clade with congeneric *C. racemosa*.

*Caulerpa lentillifera* J. Agardh is widely distributed in the tropic Indo-Pacific (Phillips et al. 1999; Schils and Coppejans 2003; Kazi et al. 2013). As an edible alga called “sea grape”, *C. lentillifera* contains multiple essential amino acids with low-level total lipid (Niwano et al. 2009). The cultivations of this species are conducted in the Philippines, Malaysia, Japan, Vietnam and China (Zemke-White and Ohno 1999; Kurashima et al. 2003; Shi 2008). In the present study, we assembled and characterized the complete chloroplast genome sequence of *C. lentillifera* (MG753774) based on the Illumina pair-end sequencing data.

The fresh thalli of *C. lentillifera* were collected from Lingtou sea area (Hainan, China; 18°68′N, 108°70′E), and were used for the total genomic DNA extraction with the modified CTAB method (Doyle and Doyle 1987). DNA samples and voucher specimens were deposited in the Marine Biological Museum of the Chinese Academy of Sciences (MBMCAS). The whole-genome sequencing was conducted with 125 bp pair-end reads on the Illumina Hiseq Platform (Illumina, San Diego, CA). In all, 686 M raw reads were obtained, and after the quality-trimmed using the software CLC Genomics Workbench v7.5 (CLC bio, Aarhus, Denmark), the resultant 643 M clean reads were then used for the cpDNA assembly using the MITObim v1.7 (Hahn et al. 2013). The complete chloroplast genome sequence annotation of *C. lentillifera* was conducted by web server DOGMA (Wyman et al. 2004). Some tRNAs, rRNAs and coding sequences were further confirmed, and manually adjusted after BLAST searches. The complete chloroplast genome of *C. lentillifera* was 119,402 bp in length. This circular chloroplast genome contains 91 protein-coding genes, 28 tRNA genes, and 3 rRNA genes. Among the tRNA genes, the tRNAMet have three copies. The content of A, C, G, and T in the chloroplast genome was 32.9%, 16.0%, 16.6%, and 34.5%, respectively. Besides, the GC content of the whole chloroplast genome was 32.6%.

A phylogenetic analysis was carried out based on concatenated dataset of 48 genes (*atp*A, *atp*B, *atp*E, *atp*H, *atp*I, *clp*P, *inf*A, *pet*A, *pet*B, *pet*G, *psa*A, *psa*B, *psa*C, *psa*J, *psb*A, *psb*C, *psb*D, *psb*E, *psb*F, *psb*H, *psb*I, *psb*J, *psb*K, *psb*L, *psb*N, *psb*T, *rbc*L, *rpl2, rpl5, rpl14, rpl16, rpl20, rpl23, rpl36, rps3, rps4, rps7, rps8, rps9, rps11, rps12, rps14, rps18, rps19, tuf*A, *ycf3, ycf4,* and *ycf12*) with a GTR + G model implemented in RAxML8.1 (Stamatakis 2014). The topology of the phylogenetic tree revealed that *C. lentillifera* clustered with congeneric *C. racemosa* (KT946602) in a monophyletic clade (Figure 1). The newly characterized *C. lentillifera* chloroplast genome will provide essential data for further studies on phylogeny and evolution of Bryopsidales and provide valuable insight conservation and restoration efforts for this economic important alga.

**Figure 1.**
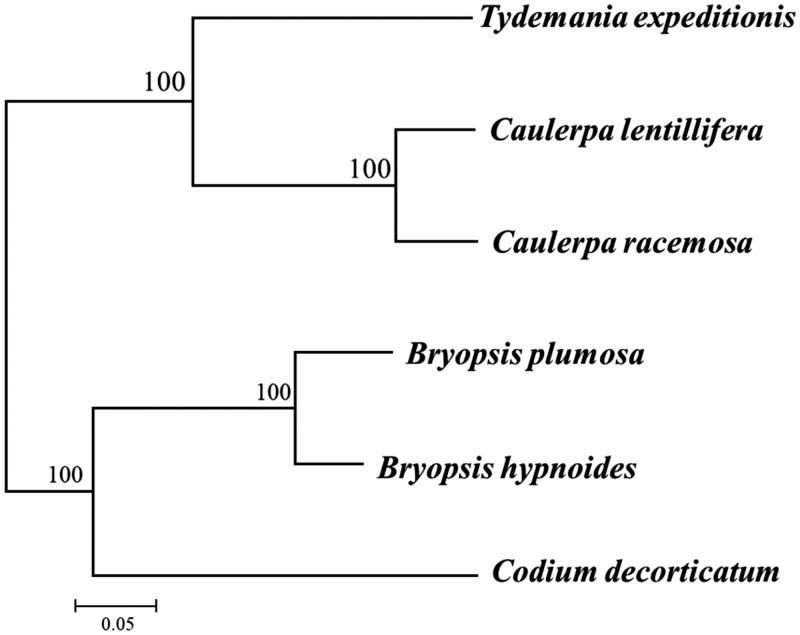
Phylogenetic tree of six species based on 48-gene nucleotide alignment from chloroplast genome. The values at the nodes correspond to bootstrap support in percentages for maximum likelihood method.
